# Indents

**Published:** 1922-02

**Authors:** 


### INDENTS

**☞Brief Articles of Technical or Scientific Value will receive notice and comment. Contributions invited.**

---

The Determination of Infection. Just as the X-ray showed that canals were seldom filled just to and not beyond the apex, so this method will show that very few root canals are perfectly sterile—yes, anywhere near sterile—before filling, and the greatest deterrent to its adoption and use is going to be that it is *too good.*

Until the present time there has been no method available to the average practitioner for testing the condition of a root canal, the practice of smelling the treatment being as futile as it is disagreeable, a laboratory test not being practical.

Dr. Gage has used this method of instantly determining whether a root canal is sterile and of estimating the degree of infection. For over six years it has been familiar to a few of his friends to whom he has shown it privately and who have used it. The chemical phase of the method has been gone into thoroughly and the soundness of it rests on a solid basis. However, it will be better to try it than to argue, so here it is:

The testing medium is di-oxygen (for experimental purposes, ordinary five-and-ten cent store peroxide of hydrogen gives good results). *Sterile* absorbent cotton, as it comes from the manufacturer, when dropped into a test tube containing this liquid immediately sinks to the bottom and *remains there.* Very slightly *infected* cotton sinks but quickly *rises again.* For example, cotton infected from around the anterior teeth may sink and remain down a short time, while that from the posterior teeth may even refuse to go down.

So delicate is the action that a wisp of cotton one end of which is infected and the other sterile, will maintain a perpendicular position in the tube, the sterile end down and the infected end up, and will rise to the surface or sink to the bottom according to whether infection or sterility predominate.

Test it as you will, the action will be found as constant as is the needle to the pole, and few and far between will be the root-canal treatments that register sterile under its delicate and exacting requirements.

An interesting series of experiments can easily be made showing the comparative efficiency of the various methods of sterilizing and incidentally you will find how very difficult it is to render anything absolutely sterile.

Another remarkable feature about the method is that a treatment, taken from a canal saturated with serum, and which you would rather expect to float, will, if it is sterile, sink and remain at the bottom.

The rule is that the longer the cotton stays down, the less infection is present—a treatment that stays down three minutes will insure you a root canal as sterile as you may practically hope to get it.

Incidentally, the method proves that di-oxygen is not a sterilizing agent (nor is peroxide of hydrogen).

Were they, the treatments immersed in them would become sterile and sink.—Dr. A. B. French, in *Oral Hygiene.*

---

JAP OBSERVATION ON DENTAL EDUCATION. Brain storm of severe intensity is now occurring in thoughts of all persons interested in education of dentists during approaching years of immediate future.

Complete insomnia may perhaps be produced by consideration of following indigestible facts:

Enormous number of ambidextrous operators is needed in U. S. A. and other arid regions to replace and repair chewing apparatus which is now unable to do so.

Authorities of exalted position make deductions of startling nature from mortality tables and other light literature which elucidate irreducible figures showing that at the end A. D. year 1922 there will be less dentists of tooth saving intentions at large in this country than at beginning of Republican year of 1920. Extreme disgust promptly registered by all honest citizens with large tooth cavities and small bank accounts when such reports are transmitted to intelligence department.

Reasons for this and other deceased conditions obtainable in dental profession are discernable if seriously investigated. At present time students must first escape from recognizable high school before being allowed to penetrate institution where dental gymnastics are imparted for cash consideration. This event seldom occurs before age of considerable magnitude has been reached; after this-Mr. Editor, must be added four entire years of elapsed time and four to six thousand dollars of evaporated money before prematurely aged person is released by faculties and examiners to perform actual tooth operations if still possible.

Reflections of Japanese school boy produce reaction of some intensity when projected against this and all similar problems of education. Boys desiring to pursue career of violin playing are not restrained by wise legislators from handling fiddle bows until comparatively safe age of twenty years has been overtaken.

Ancient game of Scottish beginnings called golf is somewhat freely indulged in by men of first, second and all intermediate childhoods, but ability to wallop ball in score equal to General Par is only developed by players who released hold on hon. nursing bottle in order to grab mid iron.

In law, Mr. Editor, practitioners who do it can be numb from collar down and still deliver one hundred per cent. efficiency of professional requirements.

Physicians can assume comfortable parking space in arm chair and diagnose diseases, whether present or not, with physical ease and financial success, but dentistry when done to human beings, instead of writing about it in text-books and picture drawings, has to be uttered through educated fingers of entire man who is able and strong enough to stand up and do so.

Brains, Mr. Editor, while almost entirely absent from many animals in human form, is extremely desirable in all dentists and should be present in sufficient quantities if possible, but unless brains when found to be present can be connected up to fingers instead of exclusively to conversation and writing abilities, results to patients often do not resemble beautiful pictures noticeable in text-books and magazines that can afford them.

Dentistry, Mr. Editor, is important occupation calling for muscles trained similar to violin fiddler or golf swatter, then why not begin by doing so at youthful age considerably before hardening of the arteries can begin to take place.

Hoping you are the same.—"Togo," in *Bulletin Illinois Dental Society.*

---

Silk Mesh for Denture Adhesion. At one time a fine gold mesh was applied to the palatal surface of rubber plates, and was considered by some of the profession to be an excellent method of plate retention, but for two reasons the success of "gold mesh plates" failed. First, because sooner or later the mesh became loose; and, second, on account of irritation, the latter being augmented by the action of the former.

Right here brings out the issue which some of my critics hold regarding plates containing a mesh surface, the contention being that all surfaces of plates must be smooth in order to avoid irritation. Without going into details at present on this subject I desire to say that a denture made following the technic here outlined, having a decidedly marked mesh surface, will cause far less irritation than any smooth or "air-chamber" denture constructed, consistent with the retaining qualities of the denture itself.

Experience covering a period of several years has convinced me that artificial dentures can be constructed with a "mesh" inner surface which will not cause undue irritation to the soft tissues of the mouth notwithstanding any statement to the contrary. Furthermore, if the evidence of satisfaction from the patients themselves is of any value, it is well to give the matter at least a consideration before passing judgment.

"Silk Mesh" Technic: The use of "silk mesh" is confined entirely to modeling compound impressions, and it is absolutely necessary to master compound technic before attempting any work involving the use of "mesh." Compound, so easy to use, is far easier to abuse and it has been condemned by many dentists. It is my opinion, however, that this is due simply to a lack of knowledge of its wonderful working qualities, plus a lack of experience that has brought on their difficulties.

After you have secured an impression in the compound (which must be subjected to all the tests of retention and stability) and have trimmed the impression to meet all the requirements of muscle freedom, you are ready to add the "mesh."

The "mesh" I am using comes in three sizes, one, one and a half and two millimeters being the diameter of the round holes.

Take a piece of the larger size "mesh" and cut to conform to the palatal surface of the impression extending to the corner line of the ridge, the remaining surface of the impression (that portion representing the buccal and labial of the model) will require the smaller size "mesh." The surface of the modeling compound is now treated lightly with chloroform, just enough should be used to make the "mesh" hold down at all points on the inside surface of the impression. This will require time and care and the patient should be dismissed and another appointment made for final adjustment with the "mesh" in position.

Now you are ready for the final setting of the impression. You will find that experience counts the most at this stage and for this reason you should get the principal of what should be done thoroughly in your mind. The object of this "mesh" addition is (when impression and tissue come into contact with each other) to force the "mesh" in two directions. This can only be accomplished by a slight softening of the inner surface of the modeling compound, using running hot water and gently and quickly setting the impression in the mouth. Caution: Do not *press too firmly*. If you have been careful, the following results will be apparent upon the impression. The hard spots in the mouth will cause almost a disappearance of the "mesh" into the compound and the soft spots will cause the "mesh" to stand well up on the surface.

Artificial stone is used to make the model, forcing it well down into all the "mesh." The separation should be done before the stone becomes entirely hardened and dry; heat is best for this, inasmuch as hot water used upon the setting stone will cause it to become softened. The reason for early separation is to enable you to pull the "mesh" from stone without leaving parts of the thread in the model.

Post-damming is rarely necessary, but by polishing down the "mesh" surface on the model where the posterior lines of plate are to rest, the effect of post-damming will be accomplished.

Time will not permit of instructions on the use of base plate, articulation and finishing, but it is just as essential to observe the details regarding the completion of your work as the taking of the impression, and while you may not have the success hoped for in your first undertaking. nevertheless, practice will soon enable you to avoid early mistakes, and when once you have observed the success of your own "mesh" undertaking, I firmly believe that you will use this method in all difficult cases.

Just one thing more and this will explain more clearly why this character of denture will not irritate the soft tissues. A "silk mesh" plate *at rest* does not lay in close *hard* contact with the tissues, the soft area of the mouth coming in contact first with the raised "mesh" on the plate tends to hold it slightly away from the hardened surfaces of the mouth, but when the full pressure of mastication comes on the denture, every circle of the "mesh" starts to perform its duty of suction and retention. Two years of handling difficult cases sent by practicing dentists are sufficient for me to recommend it to the profession.—E. Byron Kelly, in *Dental Facts* for December, 1921.

---

UP TO SIXTEEN YEARS OF AGE. If a child is brought up until the age of sixteen with a perfect set of teeth, he will in all probability go through life without much dental ills. Therefore dental education must be started early.

---

"THE AVERAGE PHYSICIAN'S ADVICE IN THE CARE OF THE TEETH OF PREGNANT WOMEN." To the Editor of the *Dental Cosmos*. Sir: I read in the September issue of the *Dental Cosmos* with surprise and amazement an article under the heading, "The Average Physician's Advice in the Care of the Teeth of Pregnant Women," by Dr. Irving S. Weinstein, of Brooklyn. I can not see why the doctor in question assumes that, because he happened to have a patient who was pregnant and was advised against treatment of her teeth, this establishes a criterion to be used in judging the average physician.

As a matter of fact, during the past ten years, at all the medical schools under the Hygiene of Pregnancy, the care of the teeth has been emphasized considerably. It is common knowledge to every student of obstetrics that during the development of the fetus the tissues of the mother are called upon to supply calcium to the fetus, and the teeth are not exempt; hence, the occurrence of caries in teeth during pregnancy. Not only is the practitioner taught to advise the filling of teeth, but even the removal of badly decayed teeth has been advocated by all leading obstetricians.

Among the many other unethical procedures, probably the most obnoxious is the custom of fee splitting. It stands to reason that when a dentist charges enough for an operation or for a certain piece of work to enable him to not only make a reasonable profit for himself, but to return to some other dentist or some other person a proportion of that fee, that he is overcharging the patient. If there is to be a rebate anywhere, in all honesty it should be given to the patient.

To offer to do a certain piece of work for a fee that is below that quoted by some other dentist, *not* because he honestly believes the lower fee to be just, but solely in order to take that patient *from some other dentist,* is equally as reprehensible as fee splitting.

The most common deviations from an ethical standard in dentistry are the following:

1. Taking advantage of the ignorance of a patient and exacting excessive fees by describing some method or procedure that sounds very difficult to the patient.

2. Encouraging the patient to believe in the success of an operation when in the nature of the case there is uncertainty.

3. Showing too little respect for the opinion of older practitioners by the younger, and offering too little encouragement to the younger practitioner by the older.

4. Speaking disparagingly of the profession of dentistry.

5. Resorting to methods of advertising and publicity that are untruthful, or that may have two interpretations, making it possible to deceive.

6. Criticizing the ability and integrity of our fellow-practitioner.

7. Fee splitting and fee cutting.—*Northwestern Journal of Dentistry.*

If Dr. Weinstein will consult Williams' "Text-Book of Obstetrics," or Dr. Lee's "Text-Book of Obstetrics," he will see the attitude of these men toward mouth hygiene of the mother during pregnancy. I am quite certain that the average physician does advise his pregnant patients to have their teeth properly attended to during pregnancy. It is inconceivable that a physician practicing medicine in this age should be responsible for the remarks attributed to him in the mentioned article.—Harry Wexler, in *Cosmos,* December, 1921.

---

Rubber Dam for Use in Fitting Porcelain Crowns. Apply rubber dam to three teeth, one on each side of tooth to be crowned, but on the tooth to be crowned force silk well under gum; slightly under, labially, if preferred. Select tooth and grind to fit and finish. Result: Field of operation clean, perfect fit, no blood and no ragged gum to, perhaps, cause gingivitis.—R. Dickson, Dubbo, *N. S. W.*

---

Preventing Shrinkage When Soldering a Bridge. The soldering of extensive bridgework involving the incisor and bicuspid teeth on each side is always attended by more or less buckling of the bridge because of shrinkage of the large body of solder used.

No investment compound will of itself entirely prevent this shrinkage.

Shrinkage can, however, be effectually controlled by use of tin foil incidental to waxing up the teeth on the cast.

Cover the incisor area of the cast with tin foil before waxing up the teeth, carrying the foil up about the sides of the abutment crowns, then when the teeth are backed up and adjusted to position this entire section of teeth between the crowns can easily be removed, separately invested and soldered and returned to its proper position on the cast.

The bridge is then invested in the usual way, and by use of little more solder the section is attached to the abutment crowns and the objectionable buckling thus overcome, the shrinkage having been confined to the section of teeth previously soldered.

I have experienced most gratifying results from this procedure and am anxious that others should profit by it. —R. V. Blake, D.M.D., in *Cosmos.*

---

To Remove Modeling Composition from Impression Trays. After having tried different methods for cleaning impression trays, I have found the following to be the most satisfactory:

Heat the impression tray in water at about 140 degrees F. and remove the bulk of the material; then immerse the tray for half a minute in boiling water. Remove and clean with a fair-sized wire brush, either by hand or on the lathe. The tray can thus be perfectly cleaned and burnished at the same time.—E. F. Boumont, in *Cosmos.*

